# Medication use in Italian nursing homes: preliminary results from the national monitoring system

**DOI:** 10.3389/fphar.2023.1128605

**Published:** 2023-05-17

**Authors:** S. Zito, E. Poluzzi, A. Pierantozzi, G. Onder, R. Da Cas, I. Ippoliti, C. Lunghi, A. Cangini, F. Trotta

**Affiliations:** ^1^ Italian Medicine Agency (AIFA), Rome, Italy; ^2^ Department of Medical and Surgical Sciences, University of Bologna, Bologna, Italy; ^3^ Department of Geriatrics, Fondazione Policlinico Universitario A. Gemelli IRCCS, Catholic University of the Sacral Heart, Rome, Italy; ^4^ National Centre for Drug Research and Evaluation, Pharmacoepidemiology Unit, Italian National Institute of Health, Rome, Italy

**Keywords:** elderly, prescribing appropriateness, quality of healthcare, medication use, nursing homes

## Abstract

**Background:** The aging population has increased concerns about the affordability, quality, and nature of long-term care for older people, emphasizing the role of nursing homes. Unlike acute hospital and primary care, there is a lack of drug consumption data in long-term care to understand regional or national healthcare policies.

**Objectives:** This study aimed to describe medication consumption by older adults and expenditure in Italian nursing homes (NHs).

**Methods:** Data on drug consumption and costs from the administrative medicine informational flows that detect medicines packages supplied to patients in health facilities and NHs were used. Data on the characteristics of the healthcare residence were from the Italian Health Ministry. Records for the year 2019, selecting the nursing homes exclusively providing elderly or mixed (elderly and disabled) were used.

**Results:** In 2019, the total expenditure on medicines in NHs amounted to 25.38 million euros, the average cost to 1.30 and the expenditure per bed to 436.18 euros. Cardiovascular drugs were the highest-consuming therapeutic class (177.0 defined daily doses—DDDs/100 days of NH stay; 22.2% of total) followed by drugs acting on the alimentary tract and metabolism (167.6% and 21.0%) and blood drugs (160.4% and 20.1%). The treatment of hypertension and heart failure was widely the most frequently used, with the consumption being driven mainly by furosemide and ramipril. Antiulcer drugs were used on average in more than half of the days of NH stay (58.5 DDDs/100 days of NH stay), representing a therapeutic category for which deprescribing initiatives are recommended. On average, almost all patients received a dose of benzodiazepines, antipsychotics and antidepressants (37.6, 35.9, and 17.7 DDDs/100 days of NH stay, respectively), confirming the high prevalence of use for these medicines. Antibiotics reached 6.8 DDDs/100 days of NH stay.

**Conclusion:** The availability of data in this specific setting allows the identification of the main interventions toward improving appropriateness and represents a challenge for drug utilization research. Data from this study suggest that proton pump inhibitors (PPIs), benzodiazepines and antibacterials can be areas of improving prescribing appropriateness.

## Introduction

The aging population has increased concerns about the affordability, quality, and nature of long-term care for older people, emphasizing the role of nursing homes, their clinical practice, and their economic sustainability (Avorn and Gurwitz, 1995; Tolson et al., 2013). Indeed, by 2050, the population in the European Union could reach 218 million people aged 60 or over, of which 1.3 million people with severe dependency in Italy alone (Pickard et al., 2007). The demand for long-term care is therefore increased by the number of older adults (aged 65 and over) suffering from multiple chronic diseases and different degrees of disability (Ouslander and Osterweil, 1994; Tolson et al., 2013). The response to this health need differs significantly from country to country and sometimes within the same state (Tolson et al., 2013) also because there is not a universally accepted definition for long-term care service or nursing home (Ribbe et al., 1997).

Assessment of older people’s pharmacological regimens in terms of appropriateness, adherence, and risk of drug interactions have too often neglected in any care setting. Residents in nursing homes are more likely to be chronically ill, with cognitive and functional impairments (Avorn and Gurwitz, 1995). These patients show age-related physiological changes that influence the pharmacokinetics and pharmacodynamics of drugs (Ruggiero et al., 2010). Since the presence of concomitant diseases and the use of multiple drugs simultaneously, the risk for potentially inappropriate drug prescription is high among these patients, with clinical consequences in terms of both adverse events and reduced benefits (Avorn and Gurwitz, 1995; Halvorsen, Selbaek, and Ruths, 2017; Tolson et al., 2013). However, nursing homes represent an ideal setting for periodic medication reviews by taking advantage of continuous professional support in monitoring healthcare status and helping medication adherence.

The Italian long-term care system for older adults is mainly based on home and residential care services provided by municipalities (for the social care part) and regions (for the healthcare/nursing-related part). According to the Italian Institut of Statistics (ISTAT), about 12,800 residential facilities existed in 2019 throughout Italy, with regional variability in the number of facilities for every 100,000 inhabitants (from 12.4% per thousand residents in Southern Italy to 31.9% in Eastern Northern Italy) (Italian Institute of Statistics - ISTAT. n.d.) Residential care in Italy is mainly delivered through nursing homes (Jessoula et al., 2018). Admission is based on healthcare needs but also income levels. The criteria for access to nursing homes and home care are quite different within the Country, depending on the region and the municipality of residence, as well as on the criteria for co-payment. Around 2.2% of the elderly subjects can access nursing homes, and about 5%–6% can access home care (Jessoula et al., 2018). In 2019 in Italy, about 300,000 beds in public nursing homes were occupied by older individuals (65 years and above) (Italian Institute of Statistics - ISTAT. n.d.).

Unlike acute care hospitals and primary care, there is no comparable data in long-term care to understand regional or national healthcare policies. While some studies on older adults in home care have been carried out in Europe, similar comparative investigations on institutional care (i.e., nursing homes - NHs) are missing (the SHELTER project et al., 2012). There are significant regional differences in the availability and organization of nursing homes in Italy according to differences in regional healthcare systems. Data on medications used in Italian nursing homes are scarce. Nursing home residents generally have more than two chronic conditions (multimorbidity (Johnston et al., 2019)). They are therefore treated with many medications (namely, polypharmacy (Sirois et al., 2019)) since there is little guidance for treating these complex patients. As reported by Onder et al. in their recent guidelines for managing older adults with multimorbidity and polypharmacy, a multidisciplinary and individualized approach is necessary, as well as the identification of those at higher risk for adverse outcomes of polypharmacy (Onder et al., 2022).

An Italian drug utilization study performed on a network of nursing homes in Northern Italy found that psychotropic medications (benzodiazepines, antipsychotics, or antidepressants), followed by proton pump inhibitors, laxatives, and antihypertensive drugs, were the most used. Mainly, psychotropics were the most commonly prescribed drugs in patients with dementia leading to a possible exacerbation of cognitive pathology, risk of serious adverse events, and drug interactions. Instead, antiulcer agents were the most widely used drugs in the cohort without cognitive disorders (L. Pasina et al., 2020a).

### Objective

This study aims to describe medication consumption by older adults and expenditures in Italian nursing homes. Describing and discussing medication consumption ad costs in nursing homes will help identify potential inappropriateness areas and define relevant monitoring and intervention approaches.

### Methods

#### Data sources

We used data on drug consumption from the “Direct and *per conto* distribution” flow. Direct distribution refers to the delivery of medications by public facilities such as Local Health Authorities or hospitals to out-of-hospital patients; these medicines belong to a defined list and are purchased by those facilities, usually at lower price. The *per conto* distribution, which means “on behalf of”, refers to the distribution of the same medicines by affiliated pharmacies; this channel is particularly used in rural areas. The information collected in the database includes the pharmaceutical service that supplied the medicine, the prescribed medicines, the supply model (direct or *per conto* distribution), costs of services (in case of *per conto* distribution), the dispensing date, the number of packages, the Anatomical Therapeutic Chemical [ATC] and the defined daily dose [DDD]. For the present analysis, data for the year 2019 were extracted, and nursing homes were selected as pharmaceutical service that supplied the medicine. Data for 2018 were also collected in order to assess stability of data flow. Among NHs providing data, only those providing elderly or mixed care (to elderly and disabled patients) were considered for the present study. Data on the characteristics of the healthcare residences were from the Italian Health Ministry. In particular, the relevant dataset contains the description, for each structure, of the number of beds, the healthcare type, and the region they belong to.

To ensure the data quality, only regions where at least 80% of the facilities regularly sent data through this flow were selected (Autonomous Provinces of Bolzano, Veneto, Friuli Venezia Giulia, Emilia Romagna, and Umbria). Instead, residences with less than five beds or with an extreme amount of Defined Daily Doses (DDDs) per bed (less than the third or more than the ninety-seventh percentile of the distribution) were excluded.

#### Data analysis

Pharmaceutical data were collected according to the Anatomical Therapeutic Chemical (ATC) classification established by the World Health Organization Collaborating Centre (WHO-CC) for Drug Statistics Methodology, and results were presented both by first and fifth ATC level. Moreover, data were analyzed according to therapeutic categories based on the ATC classification (e.g., antihypertensive, antidiabetic drugs) to perform further insights (Supplementary Table S1) (Italian Medicines Agency, 2021). Drug consumption was measured as the number of Defined Daily Doses (DDDs) (WHO Collaborating Centre for Drug Statistics Methodology, 2022).

Indicators as cost of nursing home (NH) stay per day, DDDs per 100 days of NH stay, and DDD average cost were calculated. The cost of NH stay per day is referred only to drugs expenditure. In order to assess the efficient use of resources in this setting, we also present the percentage of generics used in each therapeutic category.

The indicator DDDs/100 days of NH stay represents the number of DDDs used in a hospital nursing home divided by bed days and multiplied by 100. Analogously, the cost of NH stay per day was calculated by dividing the total expenditure by the number of days in NH provided in the reference time period. The “DDD average cost” indicator was estimated by dividing the total spending by the number of DDDs provided in the reference time period.

Analysis of antibacterial consumption (reported as total DDDs and percentage of total antibacterial consumption for each active substance) was also performed on the basis of the AWaRe classification (World Health Organization, 2019), released by the WHO to support countries' antibiotic stewardship. The Access group includes antibiotics with lower resistance potential, and the Watch group is for antibiotics at relatively high risk of selection of bacterial resistance. In contrast, antibiotics in the Reserve group should be treated as “last resort” options.

## Results

### Characteristics of the sample and overall consumption and expenditure for medications

We analysed data on drug utilisation in a sample of 802 nursing homes in five Italian Regions in 2019, accounting for a total number of 58,191 beds. They represent approximately 28.5% of the total number of beds in the Italian nursing homes (Supplementary Table S2).

**FIGURE 1:**
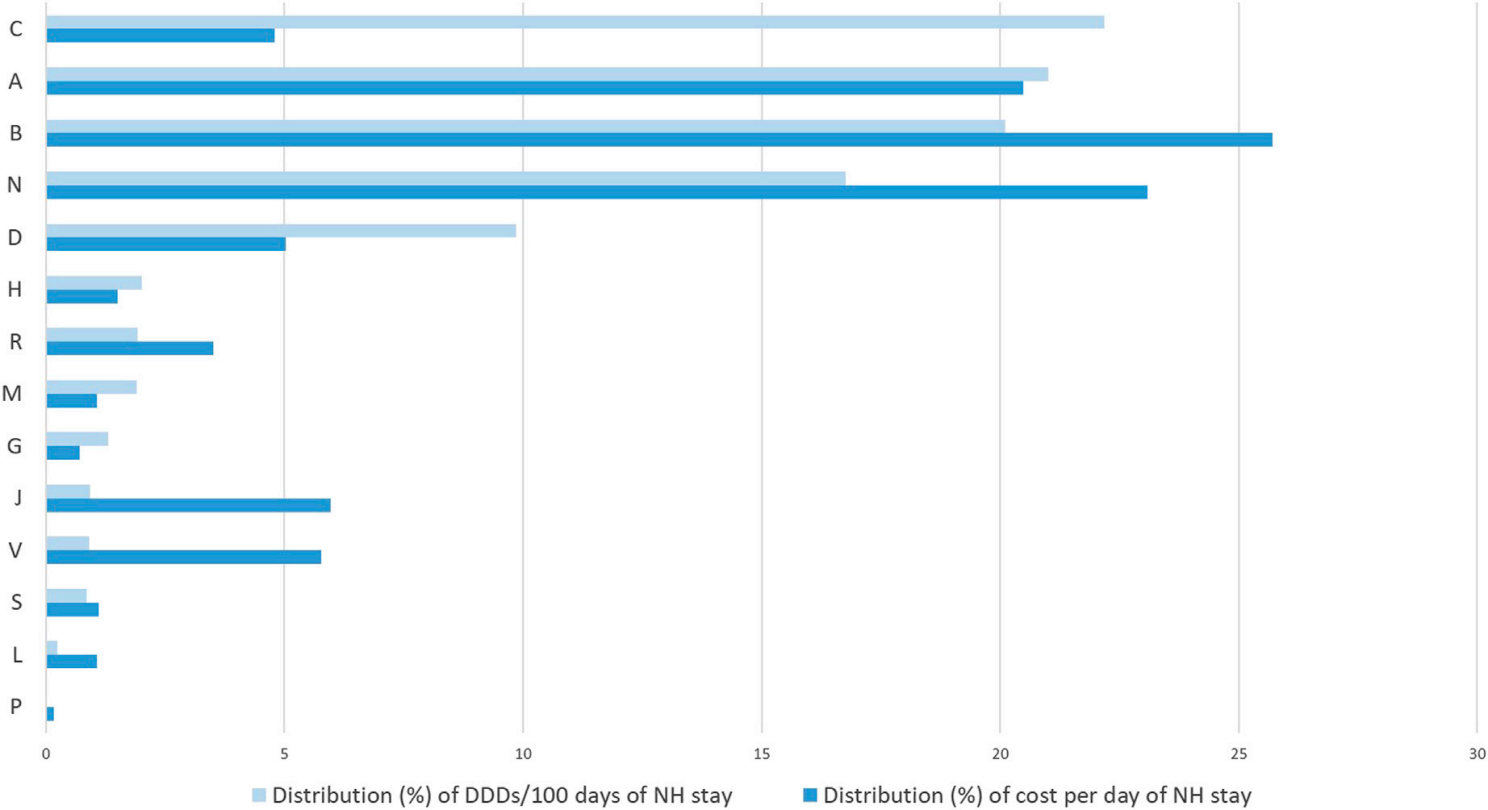
Percentage distribution (%) of DDDs/100 days of NH stay and cost (euros) per day of NH stay by ATC I level in nursing home residents (2019). DDD: defined daily dose; Anatomical Therapeutic Chemical (ATC) classification: A - alimentary tract and metabolismo; B - blood and blood forming organs; C - cardiovascular system; D - dermatologicals; G - genitourinary system and sex hormones; H - systemic hormonal preparations, excl. sex hormones and insulins; J - antiinfectives for systemic use; L - antineoplastic and immunomodulating agents; M -musculo-skeletal system; N - nervous system; P - antiparasitic products, insecticides and repellents; R - respiratory system; S - sensory organs; V - various.

Total consumption of drugs amounted to 797.86 DDDs per 100 days of NH stay. On average, the 2019 expenditure for medicines for each day of NH stay was 1.30 euros, and the total expenditure per bed was 436.18 euros. Drugs acting on the cardiovascular system (ATC: C) showed the highest consumption (177.0 DDDs/100 days of NH stay), accounting for 22.2% of all DDDs (Table 1), followed by drugs acting on the alimentary tract and metabolism (ATC: A; 167.6 DDDs/100 days of NH stay and 21.0% of total DDDs), blood drugs (ATC: B; 160.4 DDDs/100 days of NH stay and 20.1% of total DDDs), and of those acting on the central nervous system (CNS; ATC: N; 133.8 DDDs/100 days of NH stay and 16.8% of total DDDs). Blood drugs were those with the highest cost per day of NH stay (0.33 euro and 25.7% of total expenditure; Table 1; Figure 1), followed by CNS drugs (0.30 euro and 23.1% of total expenditure) and drugs acting on the alimentary tract and metabolism (0.27 euro and 20.5% of total expenditure. Antiparasitic products had the highest cost per DDD (1.11 euro), followed by anti-infective agents (1.06 euro). Nevertheless, these classes accounted only for 0.2% and 6.0% of costs per day of NH stay, respectively.

**TABLE 1:** Consumption, expenditure, and DDD average cost by ATC I level in NH residents (2019).

ATC I level	DDDs per 100 days of NH stay	%	Cost (euros) for a day of NH stay	%	DDD average cost
A	167.6	21.0	0.27	20.5	0.16
B	160.4	20.1	0.33	25.7	0.21
C	177.0	22.2	0.06	4.8	0.04
D	78.6	9.9	0.07	5.0	0.08
G	10.3	1.3	0.01	0.7	0.09
H	16.1	2.0	0.02	1.5	0.12
J	7.3	0.9	0.08	6.0	1.06
L	1.9	0.2	0.01	1.1	0.71
M	15.0	1.9	0.01	1.0	0.09
N	133.8	16.8	0.30	23.1	0.22
P	0.2	0.0	<0.005	0.2	1.11
R	15.3	1.9	0.05	3.5	0.30
S	6.9	0.9	0.01	1.1	0.21
V	7.2	0.9	0.08	5.8	1.04
**Total**	**797.6**	**100.0**	**1.3**	**100.0**	**0.16**

DDD: defined daily dose.

Anatomical Therapeutic Chemical (ATC) classification: A - alimentary tract and metabolismo; B - blood and blood forming organs; C - cardiovascular system; D - dermatologicals; G - genito urinary system and sex hormones; H - systemic hormonal preparations, excl. sex hormones and insulins; J - antiinfectives for systemic use; L - antineoplastic and immunomodulating agents; M - musculo-skeletal system; N - nervous system; P - antiparasitic products, insecticides and repellents; R - respiratory system; S - sensory organs; V - various.

Bold types are about total values.

### Medication consumption and expenditure by therapeutic class and active substance

The class with the highest consumption were the antihypertensives with 145.0 DDDs/100 days of NH stay, followed by antianemic preparations (72.9 DDDs/100 days of NH stay), drugs for constipation (71.9 DDDs/100 days of NH stay), dermatologicals (61.3 DDDs/100 days of NH stay), and drugs for peptic ulcer and gastro-esophageal reflux disease (58.5 DDDs/100 days of NH stay; Table 2).

**TABLE 2:** Consumption, expenditure and percentage of generics by therapeutic category in NH residents (2019).

Therapeutic category	DDDs/100 days of NH stay	Cost (euros) per day of NH stay	DDD average cost	% Of generic consumption	% Of generic expenditure
Antihypertensives	144.98	0.04	0.03	26.25	30.91
Antianemic preparations	72.88	0.03	0.05	0.19	0.73
Drugs for constipation	71.86	0.15	0.21	0.00	0.00
Dermatologicals	61.25	0.04	0.07	0.02	0.18
Drugs for peptic ulcer and GERD	58.50	0.02	0.03	23.54	60.52
Platelet aggregation inhibitors	43.47	0.01	0.03	79.09	62.82
Benzodiazepines	37.60	0.01	0.02	0.00	0.00
Antidepressants	35.91	0.05	0.13	34.32	17.96
Anticoagulants	30.22	0.21	0.69	0.00	0.00
Antipsychotics	17.69	0.08	0.47	55.65	21.42
Lipid-lowering agents	17.47	0.00	0.01	2.34	15.83
Antidiabetics	14.01	0.05	0.34	14.64	2.70
Blood substitutes and perfusion solutions	13.70	0.08	0.59	5.53	5.60
Agents acting on cardiovascular system	12.77	0.02	0.14	7.74	5.55
Osteoporosis drugs	11.12	0.01	0.12	19.18	17.38
Antiepileptics	9.96	0.04	0.38	27.84	19.26
Drugs for genitourinary disorders	9.19	0.01	0.07	57.61	47.51
Asthma and COPD drugs	8.69	0.04	0.45	9.07	2.40
Corticosteroids for systemic use	8.39	0.01	0.11	12.36	11.66
Antibiotics for topical use	8.37	0.01	0.16	0.00	0.00
Pain therapy	8.16	0.05	0.61	0.31	0.18
Anti-Parkinson drugs	7.82	0.04	0.49	23.09	23.20
Drugs for thyroid disorders	7.64	0.00	0.03	29.34	30.47
Drugs for gastrointestinal tract and metabolism	7.53	0.02	0.26	0.00	0.00
Antipyretics	7.06	0.01	0.17	0.00	0.00
Antibiotics	6.83	0.07	0.97	14.00	13.68
All other non-therapeutic products	6.72	0.00	0.04	0.00	0.00
Preparations inhibiting uric acid production	6.14	<0.01	0.05	9.12	9.21

DDD: defined daily dose.

Platelet aggregation inhibitors, drugs for genitourinary disorders, and antipsychotics were those with the highest utilization of generic drugs, with 79.1%, 57.6%, and 55.7%, respectively.

Anticoagulants had the highest cost per day of NH stay (0.21 euro), while antibiotics had the highest cost per DDD (0.97 euro).

Vitamin B12 (cyanocobalamin) was the substance with the highest consumption (58.98 DDDs per 100 days of NH stay; Table 3). Among the other active substances, drugs indicated for hypertension, heart failure, or nephropathies (furosemide and ramipril, 48.73 and 36.66 DDDs per 100 days of NH stay, respectively), acid-related diseases (lansoprazole, 34.56), antiplatelets (acetylsalicylic acid, 29.44) and the treatment of constipation (lactulose, 28.00) were those with the highest number of DDDs/100 days of NH stay.

**TABLE 3:** Consumption, expenditure and DDD average cost for the first 20 most used substances in NH residents (2019).

Medication	DDDs/100 days of NH stay	Cost (euros) of NH stay per day	DDD avarage cost
CYANOCOBALAMIN	58.98	0.00	182.67
FUROSEMIDE	48.73	0.01	150.93
RAMIPRIL	36.66	0.00	113.53
LANSOPRAZOLE	34.56	0.00	107.02
ACETYLSALICYLIC ACID	29.44	0.01	91.17
LACTULOSE	28.00	0.02	86.73
CHLOREXIDINE/BENZALCONIUM	22.49	0.00	69.65
ENOXAPARINA	20.95	0.10	64.88
AMLODIPINE	16.30	0.00	50.48
SODIUM HYPOCHLORITE	15.85	0.01	49.08
SODIUM CHLORIDE	14.62	0.03	45.29
SEINE	14.08	0.04	43.61
ATORVASTATIN	11.80	0.00	36.55
OMEPRAZOLE	11.64	0.01	36.05
LORAZEPAM	10.79	0.00	33.41
SODIUM PHOSPHATE	10.75	0.05	33.30
SERTRALINE	9.97	0.00	30.89
PANTOPRAZOLE	7.42	0.00	22.98
TRIAZOLAM	7.26	0.00	22.49
MACROGOL 3350/SODIUM CHLORIDE/SODIUM BICARBONATE/POTASSIUM CHLORIDE	6.92	0.02	21.44

DDD: defined daily dose.

Enoxaparin was the medication with the greatest expenditure per day of NH stay (0.10 euro), accounting for 20.95 DDDs per day. Oxygen, sodium phosphate, and seine cost 0.07, 0.05, and 0.04 euros, respectively, per day of NH stay (Table 3; Supplementary Table S5).

Table 4 shows the first 20 antibiotics by consumption in 2019 in nursing homes. Among them, the Access group accounted for 53.1% of total DDDs in 2019, while the watch group accounted for 44.6%. Amoxicillin-clavulanic acid combination (access) and ceftriaxone (watch) accounted for half of the DDDs, and among the ten most used antibiotics, seven were in the watch group.

**TABLE 4:** Consumption of the first 20 most used antibiotics in nursing home residents in 2019.

Antibiotic	ATC V level	DDDs	% Of total DDDs	2019 AWaRe classification
AMOXICILLIN/CLAVULANIC ACID	J01CR02	65,454.61	39.1	A
CEFTRIAXONE	J01DD04	19,143.50	11.4	W
CLARITHROMYCIN	J01FA09	13,631.00	8.1	W
SULFAMETHOXAZOLE/TRIMETHOPRIM	J01EE01	11,002.75	6.6	A
PIPERACILLIN/TAZOBACTAM	J01CR05	10,244.70	6.1	W
CEFIXIME	J01DD08	8,470.00	5.1	W
AZITHROMYCIN	J01FA10	7,827.00	4.7	W
MEROPENEM	J01DH02	6,090.00	3.6	W
CEFOTAXIME	J01DD01	4,630.25	2.8	W
NITROFURANTOIN	J01XE01	4,470.00	2.7	A
AMOXICILLIN	J01CA04	4,204.00	2.5	A
DOXYCYCLINE	J01AA02	2,190.00	1.3	A
FOSFOMYCIN (IV)	J01XX01	1,958.00	1.2	R
CEFTAZIDIME	J01DD02	1,402.50	0.8	W
AMIKACIN	J01GB06	1,217.50	0.7	A
NORFLOXACIN	J01MA06	1,173.00	0.7	W
VANCOMYCIN (IV)	J01XA01	993.00	0.6	W
TEICOPLANIN	J01XA02	633.50	0.4	W
MOXIFLOXACIN	J01MA14	570.00	0.3	W
CEFALEXIN	J01DB01	380.00	0.2	A
**Total** ^a^	—	**167,462.06**	**100%**	—

^a^ Total consumption for all the medications in the J01 group.

DDD: defined daily dose. 2019 AwaRe classification: A = access group; R = reserve group; W= watch group. Anatomical Therapeutic Chemical (ATC) classification.

Bold types are about total values.

As reported in Supplementary Tables S3–5, a trend in reduction of total consumption and expenditure seemed to be triggered before pandemics (−5.2% in total consumption and −1.4% in total expenditure): it was especially driven by decreasing in drugs for constipation, benzodiazepines and dermatologics. On the contrary, antipyretics and pain therapy increased (by 9.5% and 6.0%, respectively), as well as antianemic preparations, antidepressants, and antipsychotics.

## Discussion

This study is the first to analyse medication consumption in nursing homes in Italy using relevant national data flows. The availability of national monitoring on drug utilization in this specific care setting has an important adding value for the early identification of room for improvement in the quality of care offered and possible changes in practice.

### Overall consumption and expenditure for medications

We found a high pharmacological burden on nursing home residents, with an average consumption of 8 daily doses for each subject each day. Four therapeutic areas (alimentary and metabolism, blood, cardiovascular and nervous system medications) shared almost equally 80% of consumption. At the same time, expenditure was especially ascribable to three of these classes as cardiovascular medicines have a lower economic burden compared to the other classes. On the contrary, when considering drug utilization and expenditure for the general Italian older population, we noticed that adults 65 years and above were reimbursed by the Italian National Health System an average of 3.4 doses of medicines per day, of which about 50% were cardiovascular medicines (Supplementary Table S6). Cardiovascular medicines, however, accounted only for 24% of total expenditure. Moreover, while alimentary and blood medications accounted for 28% of the total consumption and nervous system medications for only 5% (Supplementary Table S6), the first two classes covered slightly less than 40% of the expenditure, followed by nervous system medications with 10%. Even if consumption and expenditure in NH residents and the older general population are not directly comparable (mostly because of different indicators and different sources of data), it seems that NH residents are exposed to a higher degree of polypharmacy than the general population and that this higher consumption might be driven by the alimentary and metabolism, blood and particularly nervous system medications. Nevertheless, as mentioned before, direct comparison is not possible. For example, while benzodiazepines are included in the nervous system medication consumption for the NH setting, this is not the case for the general older population, for which benzodiazepines are not reimbursed by the public drug plan and therefore are not accounted for in the data flow for the general population. As a consequence, nervous system medication doses are underestimated for the general older population. Still, benzodiazepines alone cannot explain the large amount of doses of nervous system medications consumed in NHs. Nursing homes are, by definition, the places where the prevalence of frailty and multimorbid older individuals go when they cannot stay at home (Damiano et al., 2022). Frailty is present in about 50% of nursing home residents, while its prevalence is between 12%–24% in community-dwelling older individuals (Kojima, 2015; O’Caoimh et al., 2021). Multimorbidity and polypharmacy can indeed increase pharmaceutical expenditures for older individuals in nursing homes, as well as the risk of potentially inappropriate prescriptions, which also cause greater costs (Caucat et al., 2020).

### Medication consumption and expenditure by therapeutic class and active substance

#### Cardiovascular medicines

In our sample, the treatment of hypertension and heart failure was widely the most frequently used, with the consumption being driven mainly by furosemide and ramipril. This finding can be thus considered consistent with the most frequent diagnoses in the older population, with evidence supporting deprescribing for these medications being still scarce (Reeve et al., 2020). The use of cardiovascular medicines in NH residents seems, in fact, similar to those of outpatients 65 and older (1.62 DDDs vs. 1.77 DDDs per subject per day), although in the nursing home population, prevention of cardiovascular events should probably be a medical need with lower priority, since subjects are more strictly monitored and with a general shorter life expectation, thus with lower impact of cardiovascular risk (e.g., for cholesterol level reduction by statins).

#### Gastrointestinal medicines

Antiulcer drugs (namely, PPIs) were used on average in more than half of the days of NH stay (58.5 DDDs/100 days of NH stay). This finding confirms the high PPIs consumption in Italian nursing homes previously reported (Pasina et al., 2020b), which enormously exceeds the use in other countries. Use of PPIs in the elderly is appropriate only for current main gastric or duodenal disorders or prevention of NSAID gastric effects. However, based on our data, NSAIDs are only rarely used, as well as the prevalence of main gastrointestinal diagnoses should be low. Moreover, differences with other countries suggest that Italian nursing homes should implement deprescribing initiatives on PPIs, taking advantage of relevant evidence from original studies and authoritative guidelines (Visser et al., 2021; Onder et al., 2022).

Laxatives are another drug class with high use and are well known for their risk of misuse or abuse (Gage et al., 2010; Gustafsson et al., 2019). Constipation and relevant laxative use may reflect physiological changes in older individuals (e.g., slower bowel motility) or be a consequence of medication use, for instance, drugs with a marked anticholinergic effect (e.g., antidepressants or antipsychotics) (Clark et al., 2010). However, chronic use of laxatives should be strongly discourdged since it can lead to adverse effects such as electrolyte imbalances and abdominal symptoms (Mounsey, Raleigh, and Wilson, 2015), with consequent worsening of health status, without adequate relief of symptoms.

#### Neuropsychiatric medicines

As for other highly consumed medications, a study comparing drug use between community-dwelling older adults and those in nursing homes in Oslo showed that older individuals were more likely to use antipsychotics, paracetamol, anxiolytics, antidepressants, and loop diuretics (Fog et al., 2019). On the other hand, antidepressants, antihypertensives, antithrombotics, calcium supplements, and vaccines could be even underused in this kind of patients (Avorn and Gurwitz, 1995).

In our study, benzodiazepines, antipsychotics, and antidepressants counted for about 90 DDDs/100 days of NH stay: on average, almost all patients receive a dose of these medicines daily. Aggregated data do not allow to distinguish single therapies with a full dose for each patient from polytherapy with low doses or even from polytherapy with higher doses for a lower percentage of patients (which are the most frequent pattern of use (Spinewine, Evrard, and Hughes, 2021)). The main reasons for using these classes could range from generic anxiety disorders and insomnia to behavioral disorders in patients with dementia. Nursing homes represent a specific setting for these diagnoses. However, benzodiazepines are considered inappropriate in older individuals by now (American Geriatrics Society Beers Criteria Update Expert Panel, 2019), which could explain the trend in decreasing use in a recent study among individuals 65 years and older in Canada (Gosselin et al., 2022). Nevertheless, in that study, the prevalence of benzodiazepine use remained high (about 30%) among older adults with at least two chronic conditions (Gosselin et al., 2022).

In nursing homes, managing residents with psychiatric or behavioral disorders with non-pharmacological treatments is, unfortunately, time- and resource-consuming. Thus, drug therapy is generally considered the most straightforward approach, especially when nursing homes suffer staff shortages (French et al., 2022). A recent study conducted in Norwegian nursing homes showed that prescription rates of psychotropic drugs such as antidepressants, antipsychotics, anxiolytics, sedatives, and hypnotics increased by almost 10% 6 months after nursing home admission (Callegari et al., 2021). Tolerance development, adverse effects, and risk of clinically significant interactions shortly challenge the sustainability of these drug therapies and can contribute to worsening older residents’ health status. This finding confirms that benzodiazepines should not be used in older adults (especially if long-term use is planned), and antipsychotics require strict monitoring of maintenance of benefits and safety profile. As for antidepressants, they would be recommended only in a minority of patients, namely, those with major depression, whereas adverse effects and interactions remain a frequent risk (National Institute for Health and Care Excellence. (2022)), and older individuals are those with the highest risk for chronic use once the treatment has been started (Lunghi et al., 2020). As a matter of fact, data from 2018 on the same sample of NHs of our study showed that benzodiazepine use was decreased by more that 10%, probably due to already ongoing initiatives on this area also in our Country drived by a positive impact of other published experiences (Supplementary Table S3).

#### Antinfectives

In our study, antibiotics did not rank in the first place because their cycles are usually short, and their cumulative amount is necessarily lower than drugs chronically used. They reached 6.8 DDDs/100 days of NH stay. The most used antibiotic medication was the combination of amoxicillin and clavulanic acid, accounting for almost 40% of total antibiotic DDDs. Although this combination is a broad-spectrum antibiotic, it is among the Access group in the 2019 AwaRe classification (World Health Organization, 2019), with lower resistance potential than antibiotics in the other groups. Ceftriaxone and clarithromycin were the second and third most used antibiotics in our sample. They are listed in the Watch group of the 2019 AwaRe classification (World Health Organization, 2019); for their potential to induce resistance, they should consider only for a limited number of cases.

The use of antibiotics in nursing homes could be attributable to preventing or treating urinary tract or respiratory infections, often without verifying the real need for treatment or identifying the best treatment option. Therefore, broad-spectrum antibiotics are preferred without a strong awareness of resistance risk. However, authoritative recommendations and real-practice studies agree that admission from a nursing home is not a sufficient condition to initiate empirical broad-spectrum antibiotics. (Goossens et al., 2005; Lopes et al., 2021).

#### Rooms for improvement

Findings from our study suggest specific areas of interventions toward an improvement of the appropriateness of drug use in Italian nursing homes. These interventions require key steps to be implemented: 1. sharing among the whole care team (clinicians, general practitioners, nurses, pharmacists) criteria to be used in the identification of the inappropriate use of a drug, 2. medication reviewing supported by digital tools, and 3. therapeutic changes, including the definition of patient’s follow-up, shared with patient and caregiver. The use of educational interventions and computerized prescription of drugs, which informatic tools may support, could stimulate this type of action and not only be applied at the nursing home level but also exported to other care settings (Lunghi et al., 2022; Crisafulli et al., 2022). A multicenter, prospective pilot study confirms that the combination of educational programs and informatic media can reduce the use of potentially inappropriate drugs in care homes (Pasina et al., 2016).

#### Strengths and limitations

The main strength of this study is that we reported recent data on medications used in nursing homes in different Italian regions. Drug use data in nursing homes are rarely available since they are not part of surveillance programs, nor are they usually monitored through electronic tools. Our data flow represents a precious resource for the surveillance of medicine consumption among institutionalized older individuals and for monitoring habits and trends of exposure to medicines in this specific setting. The final aim is to help healthcare professionals maximize safe drug therapy for the elderly while maintaining evidence-based effective treatments. Moreover, we could access consumption data on not-reimbursed medications, such as benzodiazepines, thus overcoming the inherent limitation of many healthcare databases, which detect only reimbursed services. A further limitation is the lack of diagnosis in the national data flow. This does not allow us to specifically assess the treatment appropriateness for single patients and therefore provide percentages of inappropriateness.

The DDDs/100 days of NH stay indicator is best suited to assess drug consumption in settings with institutionalized patients (including nursing homes). It allows comparisons among different areas and time trends, regardless of occupancy of the number of beds and relevant occupancy differences.

Nevertheless, this study also has some limitations. First, data collection from nursing homes has involved only a few regions, especially in northern Italy. The results are thus not representative of the entire nursing home population but only of the Italian regions included in the analysis. However, with the implementation of data collection throughout the Country, this limitation will be overcome soon, at least partially, and will allow better comparisons between different regions. When data on national and regional trends will be also available, they will represent valuable support for assessing the efficacy of local initiatives and comparing them with international experiences. The flow is nevertheless still under development. Therefore, the coverage and representativeness of Italian nursing homes are far from satisfactory. The implementation of data collection in Italian nursing homes would represent valuable input for each region to improve its specific areas of inappropriateness by striving for the best reliable standards. Time trends and comparisons among different geographical areas and types of nursing homes could be available in the following years. Moreover, even if data on drug use can be easily combined with clinical data since the population is well defined, and any health event virtually misses from monitoring initiatives, at present, our data are only aggregated and thus lack population characteristics (e.g., socio-demographic information, indication of use, comorbidity burden, and clinical outcomes). Moreover, a large heterogeneity exists between different nursing homes in the management of medication allocation. As an example, in some nursing homes medications are supplied directly by local health authorities, while in others they are supplied through general practitioner. Consequently, no stratification on age group, gender, or condition can be performed. Finally, the varying regional availability of beds in the nursing home could result in a selection of the population accessing these facilities.

## Conclusion

Monitoring drug therapy in nursing homes represents a challenge for drug utilization research. Improving the quality of healthcare for older patients is one of the main goals of high-middle income countries. The availability of data on drug use in this specific setting allows the identification of the main therapeutic areas needing interventions and the assessment of their consequences. In Italy, cardiovascular medicines, followed by antiulcer and laxative agents, and drugs used for psychiatric disorders were the most used. Most of these medications, especially PPIs and benzodiazepines, together with broad-spectrum antibacterials, can be the target of quality improvement initiatives, as suggested by the relevant Italian recommendations.

## Data Availability

The datasets generated during and/or analysed during the current study are not publicly available because of data sharing legal restrictions, the dataset including individual records cannot be made publicly available. However, aggregated data will be shared on reasonable request to the corresponding author.
