# Supplementary material for: Medication use in Italian nursing homes: preliminary results from the national monitoring system
Source: Front Pharmacol. 2023 May 17;14:1128605. doi: 10.3389/fphar.2023.1128605 (PMC10229842; doi:10.3389/fphar.2023.1128605)
Supplement: Supplementary file 1 [file DataSheet3.docx]

**Supplementary Material**

**Medication use in Italian nursing homes: preliminary results from the National monitoring system**

**Zito S., Poluzzi E.*, Pierantozzi A., Onder G., Da Cas R., Ippoliti I., Lunghi C., Cangini A., Trotta F.**

***Correspondence:**

Elisabetta Poluzzi

elisabetta.poluzzi@unibo.it

**Table S1. Therapeutic categories and Anatomical Therapeutic Chemical (ATC)** **classification**

| **Antihypertensives** | C02AB01 C02AC01 C02AC05 C02CA C02CA04 C02CA06 C03AA03 C03BA04 C03BA08 C03BA11 C03CA01 C03CA03 C03CA04 C03CC01 C03DA01 C03DA02 C03DA03 C03DA04 C03EA01 C03EA14 C03EB01 C07AA03 C07AA05 C07AA06 C07AA07 C07AA12 C07AB02 C07AB03 C07AB04 C07AB05 C07AB07 C07AB08 C07AB09 C07AB12 C07AB14 C07AG01 C07AG02 C07BB07 C07BB12 C07CA02 C07CB02 C07CB03 C08CA01 C08CA02 C08CA03 C08CA04 C08CA05 C08CA06 C08CA07 C08CA08 C08CA09 C08CA11 C08CA12 C08CA13 C08CA16 C08DA01 C08DA02 C08DB01 C09AA01 C09AA02 C09AA03 C09AA04 C09AA05 C09AA06 C09AA07 C09AA08 C09AA09 C09AA10 C09AA11 C09AA12 C09AA13 C09AA15 C09BA01 C09BA02 C09BA03 C09BA04 C09BA05 C09BA06 C09BA07 C09BA08 C09BA09 C09BA12 C09BA13 C09BA15 C09BB02 C09BB04 C09BB05 C09BB07 C09BB12 C09BX01 C09BX02 C09CA01 C09CA02 C09CA03 C09CA04 C09CA06 C09CA07 C09CA08 C09DA01 C09DA02 C09DA03 C09DA04 C09DA06 C09DA07 C09DA08 C09DB01 C09DB02 C09DB07 C09DX03 C09DX04 C09XA02 C09XA52 |
| --- | --- |
| **Antianemic preparation** | B03AA01 B03AA03 B03AA07 B03AB B03AB02 B03AB05 B03AB08 B03AB09 B03AC B03AC01 B03AD B03AD03 B03BA01 B03BA03 B03BA04 B03BA51 B03BA53 B03BB01 B03XA01 B03XA02 B03XA03 |
| **Drugs for constipation** | A06AA01 A06AB02 A06AB05 A06AB06 A06AB08 A06AB20 A06AB56 A06AB57 A06AB58 A06AC01 A06AC03 A06AC08 A06AC51 A06AD A06AD10 A06AD11 A06AD12 A06AD15 A06AD17 A06AD61 A06AD65 A06AG A06AG01 A06AG04 A06AG10 A06AG11 A06AG20 A06AH01 A06AH03 A06AH05 A06AX01 A06AX04 A06AX05 |
| **Dermatologicals** | D02AB D02AX D02BB02 D03AX D03AX01 D03AX05 D03BA D03BA03 D03BA52 D04AA D04AA10 D04AA13 D04AA22 D04AA32 D04AB D04AB01 D04AB04 D04AX D05AX02 D05AX03 D05AX04 D05AX05 D05AX52 D05BA02 D05BB02 D06BA01 D06BA51 D06BB04 D06BB10 D06BB12 D06BX01 D06BX02 D08AC02 D08AC52 D08AD D08AE03 D08AG02 D08AG03 D08AJ D08AJ01 D08AJ03 D08AJ05 D08AJ06 D08AK04 D08AL30 D08AX D08AX01 D08AX02 D08AX04 D08AX06 D08AX07 D11AE D11AF D11AH01 D11AH02 D11AH04 D11AH05 D11AX D11AX01 D11AX10 D11AX16 D11AX18 D11AX21 D11AX22 D11AX24 |
| **Drugs for peptic ulcer and GERD** | A02AA04 A02AD01 A02AD02 A02AF02 A02AH A02BA01 A02BA02 A02BA03 A02BA04 A02BA06 A02BA53 A02BB01 A02BC01 A02BC02 A02BC03 A02BC04 A02BC05 A02BX02 A02BX05 A02BX08 A02BX13 |
| **Platelet aggregation inhibitors excl. heparin** | B01AC B01AC02 B01AC03 B01AC04 B01AC05 B01AC06 B01AC07 B01AC09 B01AC10 B01AC11 B01AC13 B01AC16 B01AC17 B01AC18 B01AC21 B01AC22 B01AC23 B01AC24 B01AC25 B01AC27 B01AC30 B01AC56 |
| **Benzodiazepines** | N05BA N05BA01 N05BA02 N05BA04 N05BA05 N05BA06 N05BA08 N05BA09 N05BA10 N05BA11 N05BA12 N05BA14 N05BA16 N05BA19 N05BA21 N05CD01 N05CD02 N05CD03 N05CD04 N05CD05 N05CD06 N05CD08 N05CD09 N05CF01 N05CF02 N05CF03 |
| **Antidepressant** | N06AA02 N06AA04 N06AA06 N06AA09 N06AA10 N06AA16 N06AA21 N06AB03 N06AB04 N06AB05 N06AB06 N06AB08 N06AB10 N06AF03 N06AX01 N06AX03 N06AX05 N06AX11 N06AX12 N06AX16 N06AX18 N06AX21 N06AX22 N06AX25 N06AX26 N06AX27 N06CA N06CA01 |
| **Anticoagulants** | B01AA03 B01AA07 B01AB B01AB01 B01AB02 B01AB04 B01AB05 B01AB06 B01AB07 B01AB08 B01AB11 B01AB12 B01AD02 B01AD04 B01AD11 B01AD12 B01AE02 B01AE03 B01AE06 B01AE07 B01AF01 B01AF02 B01AF03 B01AX01 B01AX04 B01AX05 B01AX07 |
| **Antipsychotics** | N05AA01 N05AA02 N05AA03 N05AB02 N05AB03 N05AB06 N05AC01 N05AD01 N05AD06 N05AD08 N05AE04 N05AE05 N05AF05 N05AG02 N05AH01 N05AH02 N05AH03 N05AH04 N05AH05 N05AH06 N05AL01 N05AL03 N05AL05 N05AN01 N05AX08 N05AX12 N05AX13 N05AX15 N05AX16 |
| **Lipid-lowering agents** | C10AA01 C10AA02 C10AA03 C10AA04 C10AA05 C10AA07 C10AA08 C10AB02 C10AB04 C10AB05 C10AX06 C10AX09 C10AX12 C10AX13 C10AX14 C10BA02 C10BA04 C10BA06 C10BX06 C10BX09 C10BX11 |
| **Antidiabetics** | A10AB01 A10AB04 A10AB05 A10AB06 A10AC01 A10AC04 A10AD01 A10AD04 A10AD05 A10AE04 A10AE05 A10AE06 A10AE54 A10AE56 A10BA02 A10BB01 A10BB02 A10BB07 A10BB08 A10BB09 A10BB12 A10BD01 A10BD02 A10BD03 A10BD04 A10BD05 A10BD06 A10BD07 A10BD08 A10BD09 A10BD10 A10BD11 A10BD13 A10BD15 A10BD16 A10BD19 A10BD20 A10BD21 A10BD23 A10BF01 A10BG02 A10BG03 A10BH01 A10BH02 A10BH03 A10BH04 A10BH05 A10BJ01 A10BJ02 A10BJ03 A10BJ05 A10BJ06 A10BK01 A10BK02 A10BK03 A10BK04 A10BX02 |
| **Blood substitutes and perfusion solutions** | A10AB01 A10AB04 A10AB05 A10AB06 A10AC01 A10AC04 A10AD01 A10AD04 A10AD05 A10AE04 A10AE05 A10AE06 A10AE54 A10AE56 A10BA02 A10BB01 A10BB02 A10BB07 A10BB08 A10BB09 A10BB12 A10BD01 A10BD02 A10BD03 A10BD04 A10BD05 A10BD06 A10BD07 A10BD08 A10BD09 A10BD10 A10BD11 A10BD13 A10BD15 A10BD16 A10BD19 A10BD20 A10BD21 A10BD23 A10BF01 A10BG02 A10BG03 A10BH01 A10BH02 A10BH03 A10BH04 A10BH05 A10BJ01 A10BJ02 A10BJ03 A10BJ05 A10BJ06 A10BK01 A10BK02 A10BK03 A10BK04 A10BX02 |
| **Agents acting on cardiovascular system** | C01AA05 C01AA08 C01BA02 C01BA03 C01BA13 C01BB02 C01BC03 C01BC04 C01BD01 C01BD05 C01BD07 C01CA01 C01CA02 C01CA03 C01CA04 C01CA06 C01CA07 C01CA17 C01CA19 C01CA24 C01CA26 C01CE03 C01CX08 C01DA02 C01DA05 C01DA08 C01DA14 C01DX11 C01EA01 C01EB07 C01EB09 C01EB10 C01EB15 C01EB16 C01EB17 C01EB18 C01EB21 C04AD03 C04AX C04AX21 |
| **Osteoporosis drugs** | A02AC01 A11CC03 A11CC04 A11CC05 A11CC06 A12AA03 A12AA04 A12AA20 A12AX G03XC01 G03XC02 G03XC05 H05AA02 M05BA01 M05BA02 M05BA03 M05BA04 M05BA06 M05BA07 M05BA08 M05BB03 M05BX01 M05BX03 M05BX04 M05BX05 |
| **Antiepilectics** | N03AA02 N03AA03 N03AA04 N03AB02 N03AB52 N03AD01 N03AE01 N03AF01 N03AF02 N03AF03 N03AF04 N03AG N03AG01 N03AG02 N03AG03 N03AG04 N03AG06 N03AX03 N03AX09 N03AX10 N03AX11 N03AX14 N03AX15 N03AX17 N03AX18 N03AX21 N03AX22 N03AX23 |
| **Drugs for genitourinary disorders** | G04BD02 G04BD04 G04BD06 G04BD07 G04BD08 G04BD09 G04BD11 G04BD12 G04CA G04CA01 G04CA02 G04CA03 G04CA04 G04CA52 G04CA53 G04CB01 G04CB02 G04CX G04CX01 G04CX02 G04CX03 |
| **Asthma and COPD drugs** | R03AC02 R03AC03 R03AC04 R03AC12 R03AC13 R03AC18 R03AC19 R03AK R03AK03 R03AK04 R03AK06 R03AK07 R03AK08 R03AK10 R03AK11 R03AK13 R03AL01 R03AL02 R03AL03 R03AL04 R03AL05 R03AL06 R03AL08 R03AL09 R03BA01 R03BA02 R03BA03 R03BA05 R03BA07 R03BA08 R03BB01 R03BB02 R03BB04 R03BB05 R03BB06 R03BB07 R03BC01 R03BC03 R03CC02 R03CC13 R03DA R03DA01 R03DA04 R03DA05 R03DA08 R03DA11 R03DC01 R03DC03 R03DX05 R03DX07 R03DX09 R03DX10 |
| **Corticosteroids for systemic use** | H02AB01 H02AB02 H02AB04 H02AB06 H02AB07 H02AB08 H02AB09 H02AB10 H02AB13 H02BX H02BX01 |
| **Antibiotics for topical use** | A07AA02 A07AA06 A07AA09 A07AA11 A07AA12 A07AA51 D06AA D06AA02 D06AX01 D06AX04 D06AX07 D06AX09 D06AX12 D06AX13 D06AX14 D06C D10AF01 D10AF02 D10AF04 D10AF05 D10AF51 D10AF52 G01AA05 G01AA09 G01AA10 G01AF20 R01AX06 R01AX10 S01AA01 S01AA02 S01AA11 S01AA12 S01AA13 S01AA23 S01AA26 S01AA27 S01AA30 S01AE01 S01AE02 S01AE03 S01AE05 S01AE07 S01AX11 S02AA S02AA15 S02AA30 S03AA30 |
| **Pain therapy** | N01AH03 N02AA01 N02AA03 N02AA05 N02AA55 N02AA59 N02AB02 N02AB03 N02AD01 N02AE01 N02AG01 N02AJ06 N02AJ08 N02AJ13 N02AJ14 N02AJ17 N02AX02 N02AX06 N02BE51 N03AX12 N03AX16 |
| **Antiparkinson drugs** | N04AA01 N04AA02 N04AA03 N04AA11 N04AB02 N04BA02 N04BA03 N04BA05 N04BB01 N04BC01 N04BC02 N04BC04 N04BC05 N04BC06 N04BC07 N04BC08 N04BC09 N04BD01 N04BD02 N04BD03 N04BX01 N04BX02 N04BX04 |
| **Drugs for thyroid disorders** | H03AA01 H03AA02 H03AA03 H03AA05 H03BB02 H03BB52 H03BC01 H03BX02 |
| **Drugs for gastrointestinal tract** | A02AX A02X A09AA02 A09AA04 A11AA03 A11BA A11CA01 A11CB A11CC55 A11DA01 A11DB A11EA A11GA01 A11HA01 A11HA02 A11HA03 A11HA05 A11HA08 A11HA30 A11HA32 A11JA A11JB A12AX A12BA A12BA01 A12BA30 A12CB01 A12CC08 A12CX A13A A14AB01 |
| **Antipyretics** | N02BA01 N02BA16 N02BA51 N02BB02 N02BB54 N02BB74 N02BE01 N02BE51 |
| **Antibiotics** | A02BD08 J01AA02 J01AA04 J01AA05 J01AA07 J01AA08 J01AA12 J01BA01 J01BA02 J01CA01 J01CA04 J01CA06 J01CA12 J01CE01 J01CE08 J01CF04 J01CF05 J01CR01 J01CR02 J01CR04 J01CR05 J01DB01 J01DB03 J01DB04 J01DB05 J01DB07 J01DC01 J01DC02 J01DC03 J01DC04 J01DC06 J01DC09 J01DC10 J01DD01 J01DD02 J01DD04 J01DD07 J01DD08 J01DD09 J01DD12 J01DD13 J01DD14 J01DD16 J01DD52 J01DE01 J01DF01 J01DH02 J01DH03 J01DH04 J01DH51 J01DI01 J01DI02 J01DI54 J01EC02 J01EE01 J01FA01 J01FA02 J01FA06 J01FA07 J01FA09 J01FA10 J01FA11 J01FA12 J01FA14 J01FA15 J01FF01 J01FF02 J01GA01 J01GB01 J01GB03 J01GB06 J01GB07 J01MA01 J01MA02 J01MA03 J01MA04 J01MA06 J01MA07 J01MA10 J01MA12 J01MA14 J01MA17 J01MB04 J01MB06 J01XA01 J01XA02 J01XA04 J01XB01 J01XD01 J01XE01 J01XX01 J01XX03 J01XX08 J01XX09 J01XX11 |
| **All other non-therapeutic products** | B05CB01 V07AB V07AC |
| **Preparation inhibiting uric acid production** | M04AA01 M04AA03 M04AB05 M04AC01 M04AX |

**Table S2. Number of NHs and beds**

| **Region** | **Total number of NHs (%)** | | **Number of beds (%)** | |
| --- | --- | --- | --- | --- |
|  | **2018** | **2019** | **2018** | **2019** |
| Bolzano Province | 66 (8.2) | 66 (8.2) | 3,673 (6.4) | 3,689 (6.3) |
| Veneto | 329 (40.9) | 334 (41.7) | 30,839 (53.5) | 31,113 (53.5) |
| Friuli Venezia Giulia | 73 (9.1) | 67 (8.4) | 6,651 (11.5) | 6,689 (11.5) |
| Emilia-Romagna | 299 (37.2) | 296 (36.9) | 15,157 (26.3) | 15,334 (26.4) |
| Umbria | 37 (4.6) | 39 (4.9) | 1,363 (2.4) | 1,366 (2.4) |
| **Total** | **804** | **802** | **57,683** | **58,191** |

Data are from The Medicines Utilisation Monitoring Centre (2021). National Report on Medicines Use in Older Adults in Italy, 2021. Year 2019. Rome: Italian Medicines Agency AIFA; https://www.aifa.gov.it/documents/20142/1577699/OsMed_Farmaci_anziani_13.10.2021.pdf. Last access: March 07, 2023

**Table S3.** Differences in consumption and expenditure for medicines in NH residents by therapeutic category between 2019 and 2018

| **Therapeutic category** | **DDDs/100 days of NH stay** | | **Cost per day of NH stay** | | **DDD average cost** | |
| --- | --- | --- | --- | --- | --- | --- |
|  | **2019** | $\boldsymbol{\Delta}$**% 19-18** | **2019** | $\boldsymbol{\Delta}$**% 19-18** | **2019** | $\boldsymbol{\Delta}$**% 19-18** |
| Antihypertensives | 144.98 | 0.36 | 0.04 | -2.96 | 0.03 | -3.51 |
| Antianemic preparations | 72.88 | 8.25 | 0.03 | -5.28 | 0.05 | -12.68 |
| Drugs for constipation | 71.86 | -19.99 | 0.15 | 3.10 | 0.21 | 28.59 |
| Dermatologicals | 61.25 | -22.72 | 0.04 | 3.07 | 0.07 | 33.10 |
| Drugs for peptic ulcer and GERD | 58.50 | -1.33 | 0.02 | -1.92 | 0.03 | -0.80 |
| Platelet aggregation inhibitors | 43.47 | -6.31 | 0.01 | -6.75 | 0.03 | -0.68 |
| Benzodiazepines | 37.60 | -10.81 | 0.01 | -6.17 | 0.02 | 4.99 |
| Antidepressants | 35.91 | 3.71 | 0.05 | 4.98 | 0.13 | 1.02 |
| Anticoagulants | 30.22 | -2.80 | 0.21 | -9.01 | 0.69 | -6.58 |
| Antipsychotics | 17.69 | 2.81 | 0.08 | 3.78 | 0.47 | 0.73 |
| Lipid-lowering agents | 17.47 | 6.54 | 0.00 | -14.25 | 0.01 | -19.69 |
| Antidiabetics | 14.01 | -5.88 | 0.05 | -7.33 | 0.34 | -1.75 |
| Blood substitutes and perfusion solutions | 13.70 | -2.30 | 0.08 | 1.90 | 0.59 | 4.09 |
| Agents acting on cardiovascular system | 12.77 | -10.96 | 0.02 | -1.92 | 0.14 | 9.92 |
| Osteoporosis drugs | 11.12 | -0.87 | 0.01 | -1.07 | 0.12 | -0.41 |
| Antiepileptics | 9.96 | -13.95 | 0.04 | 3.08 | 0.38 | 19.54 |
| Drugs for genitourinary disorders | 9.19 | 1.95 | 0.01 | -1.83 | 0.07 | -3.91 |
| Asthma and COPD drugs | 8.69 | 2.21 | 0.04 | 1.54 | 0.45 | -0.86 |
| Corticosteroids for systemic use | 8.39 | -0.02 | 0.01 | -19.21 | 0.11 | -19.36 |
| Antibiotics for topical use | 8.37 | 2.66 | 0.01 | 16.03 | 0.16 | 12.79 |
| Pain therapy | 8.16 | 5.98 | 0.05 | -15.40 | 0.61 | -20.34 |
| Anti-Parkinson drugs | 7.82 | -7.78 | 0.04 | 9.28 | 0.49 | 18.25 |
| Drugs for thyroid disorders | 7.64 | 2.28 | 0.00 | 19.96 | 0.03 | 17.04 |
| Drugs for gastrointestinal tract and metabolism | 7.53 | -7.29 | 0.02 | 6.67 | 0.26 | 14.81 |
| Antipyretics | 7.06 | 9.54 | 0.01 | -0.19 | 0.17 | -9.08 |
| Antibiotics | 6.83 | -1.96 | 0.07 | -8.70 | 0.97 | -7.07 |
| All other non-therapeutic products | 6.72 | -34.14 | 0.00 | 4.62 | 0.04 | 58.53 |
| Preparations inhibiting uric acid production | 6.14 | 2.07 | <0.01 | 19.97 | 0.05 | 17.29 |

DDD: defined daily dose.

**Table S4.** Differences in consumption and expenditure for the first 20 most used substances in NH residents between 2019 and 2018

| **Substances** | **DDDs/100 days**  **of NH stay** | | **Cost per day of NH stay** | | **DDD average cost** | |
| --- | --- | --- | --- | --- | --- | --- |
|  | **2019** | $\boldsymbol{\Delta}$**% 19-18** | **2019** | $\boldsymbol{\Delta}$**% 19-18** | **2019** | $\boldsymbol{\Delta}$**% 19-18** |
| CYANOCOBALAMIN | 58.98 | 4.86 | 0.00 | 4.73 | 182.67 | -0.33 |
| FUROSEMIDE | 48.73 | 2.62 | 0.01 | -12.12 | 150.93 | -14.54 |
| RAMIPRIL | 36.66 | 4.80 | 0.00 | -80.03 | 113.53 | -80.98 |
| LANSOPRAZOLE | 34.56 | 5.31 | 0.00 | -97.52 | 107.02 | -97.65 |
| ACETYLSALICYLIC ACID | 29.44 | -10.24 | 0.01 | -7.94 | 91.17 | 2.35 |
| LACTULOSE | 28.00 | 2.60 | 0.02 | 9.24 | 86.73 | 6.25 |
| CHLOREXIDINE/BENZALCONIUM | 22.49 | -43.09 | 0.00 | -3.92 | 69.65 | 68.49 |
| ENOXAPARINA | 20.95 | -8.43 | 0.10 | -27.74 | 64.88 | -21.26 |
| AMLODIPINE | 16.30 | 2.62 | 0.00 | 1,024.82 | 50.48 | 993.79 |
| SODIUM HYPOCHLORITE | 15.85 | -3.97 | 0.01 | 3.11 | 49.08 | 7.15 |
| SODIUM CHLORIDE | 14.62 | -19.11 | 0.03 | 0.61 | 45.29 | 24.13 |
| SEINE | 14.08 | -18.12 | 0.04 | 6.81 | 43.61 | 30.18 |
| ATORVASTATIN | 11.80 | 15.13 | 0.00 | 53.56 | 36.55 | 33.10 |
| OMEPRAZOLE | 11.64 | 1.64 | 0.01 | 10.61 | 36.05 | 8.60 |
| LORAZEPAM | 10.79 | 9.71 | 0.00 | 29.82 | 33.41 | 18.08 |
| SODIUM PHOSPHATE | 10.75 | -53.40 | 0.05 | -1.22 | 33.30 | 111.52 |
| SERTRALINE | 9.97 | 6.24 | 0.00 | 239.60 | 30.89 | 218.99 |
| PANTOPRAZOLE | 7.42 | -20.56 | 0.00 | 14.15 | 22.98 | 43.40 |
| TRIAZOLAM | 7.26 | 4.44 | 0.00 | -98.08 | 22.49 | -98.16 |
| MACROGOL 3350/SODIUM CHLORIDE/SODIUM BICARBONATE/POTASSIUM CHLORIDE | 6.92 | 3.90 | 0.02 | 5.11 | 21.44 | 1.17 |

DDD: defined daily dose.

**Table S5.** Differences in consumption and expenditure in NH residents for the 20 more expensive substances between 2019 and 2018

| **Substances** | **DDDs/100 days of NH stay** | | **cost per day of NH stay** | | **DDD average cost** | |
| --- | --- | --- | --- | --- | --- | --- |
|  | **2019** | $\boldsymbol{\Delta}$**% 19-18** | **2019** | $\boldsymbol{\Delta}$**% 19-18** | **2019** | $\boldsymbol{\Delta}$**% 19-18** |
| ENOXAPARIN | 20.95 | -8.43 | 0.10 | -27.74 | 64.88 | -21.26 |
| OXYGEN | n.a. | - | 0.07 | 19.18 | n.a. | - |
| SODIUM PHOSPHATE | 10.75 | -53.40 | 0.05 | -1.22 | 33.30 | 111.52 |
| SEINE | 14.08 | -18.12 | 0.04 | 6.81 | 43.61 | 30.18 |
| APIXABAN | 1.44 | 47.32 | 0.03 | 23.54 | 4.45 | -16.32 |
| TRAZODONE | 6.59 | 7.63 | 0.03 | 8.55 | 20.42 | 0.64 |
| SODIUM CHLORIDE | 14.62 | -19.11 | 0.03 | 0.61 | 45.29 | 24.13 |
| PROMAZINE | 1.84 | 4.12 | 0.03 | 12.29 | 5.69 | 7.62 |
| RIVAROXABAN | 1.33 | 34.19 | 0.03 | 25.95 | 4.12 | -6.33 |
| OXYCODONE/NALOXONE | 0.54 | 9.54 | 0.02 | 1.60 | 1.66 | -7.44 |
| AMINO ACIDS/ELECTROLYTES/GLUCOSE/LIPIDS | 0.19 | 15.95 | 0.02 | 1.03 | 0.58 | -13.05 |
| INSULIN GLARGINE | 1.94 | -0.74 | 0.02 | -2.25 | 6.00 | -1.72 |
| MACROGOL 3350/SODIUM CHLORIDE/SODIUM BICARBONATE/ POTASSIUM CHLORIDE | 6.92 | 3.90 | 0.02 | 5.33 | 21.44 | 1.17 |
| PIPERACILLIN/TAZOBACTAM | 0.21 | 13.16 | 0.02 | 28.39 | 0.64 | 13.22 |
| LACTULOSE | 28.01 | 2.63 | 0.02 | 9.27 | 86.75 | 6.25 |
| LEVETIRACETAM | 3.48 | 3.67 | 0.02 | -7.93 | 10.78 | -11.38 |
| EDOXABAN | 0.62 | 95.03 | 0.02 | 83.61 | 1.91 | -6.05 |
| LEVODOPA/CARBIDOPA | 3.49 | -7.26 | 0.01 | 32.59 | 10.80 | 42.66 |
| QUETIAPINE | 4.62 | 6.93 | 0.01 | -12.34 | 14.29 | -18.19 |
| SILVER SULFADIAZINE | 5.85 | 2.07 | 0.01 | 8.92 | 18.12 | 6.49 |

DDD: defined daily dose. n.a.: not applicable.

Δ% 19-18: Relative difference between 2019 and 2018.

**Table S6.** Consumption by ATC I level in general population aged ≥65 (2019)

| **ATC I level** | **DDDs/1000 inhabitants per day** | **%** | **Yearly expenditure *per capita* (euros)** | **%** | **DDD average cost** |
| --- | --- | --- | --- | --- | --- |
| A | 498 | 14.8 | 134 | 20.3 | 0.10 |
| B | 420 | 12.5 | 127 | 19.3 | 0.11 |
| C | 1616 | 48.1 | 158 | 24.0 | 0.04 |
| D | 12 | 0.4 | 3 | 0.5 | 0.09 |
| G | 318 | 9.5 | 20 | 3.0 | 0.02 |
| H | 51 | 1.5 | 17 | 2.6 | 0.12 |
| J | 27 | 0.8 | 24 | 3.6 | 0.32 |
| L | 19 | 0.6 | 29 | 4.4 | 0.56 |
| M | 92 | 2.7 | 20 | 3.0 | 0,08 |
| N | 130 | 3.9 | 65 | 9.9 | 0.18 |
| P | 1 | 0.0 | 0 | - | - |
| R | 107 | 3.2 | 11 | 1.7 | 0.04 |
| S | 73 | 2.2 | 13 | 2.0 | 0.07 |
| V | 1 | 0.0 | 7 | 1.1 | 2.56 |
| **Total** | **3363** | **100** | **659** | **100** | **0.07** |

DDD: defined daily dose.

Anatomical Therapeutic Chemical (ATC) classification: A - alimentary tract and metabolismo; B - blood and blood forming organs; C - cardiovascular system; D - dermatologicals; G - genito urinary system and sex hormones; H - systemic hormonal preparations, excl. sex hormones and insulins; J - antiinfectives for systemic use; L - antineoplastic and immunomodulating agents; M - musculo-skeletal system; N - nervous system; P - antiparasitic products, insecticides and repellents; R - respiratory system; S - sensory organs; V - various.

Data are from The Medicines Utilisation Monitoring Centre (2021). National Report on Medicines Use in Older Adults in Italy, 2021. Year 2019. Rome: Italian Medicines Agency AIFA; https://www.aifa.gov.it/documents/20142/1577699/OsMed_Farmaci_anziani_13.10.2021.pdf. Last access: March 7, 2023.

**Table S7.** Consumption of the first 20 most used substances in general population aged ≥65 in 2019

| **Substances** | **DDDs/1000 inhabitants per day** |
| --- | --- |
| ACETYLSALICYLIC ACID | 276.90 |
| RAMIPRIL | 278.70 |
| ATORVASTATIN | 224.10 |
| FUROSEMIDE | 138.60 |
| AMLODIPINE | 131.00 |
| PANTOPRAZOLE | 106.40 |
| METFORMIN | 97.50 |
| OMEPRAZOLE | 75.90 |
| LANSOPRAZOLE | 68.30 |
| SIMVASTATIN | 64.50 |
| LEVOTHYROXINE | 51.00 |
| ROSUVASTATINE | 60.50 |
| NEBIVOLOLO | 53.60 |
| TAMSULOSINE | 76.00 |
| CLOPIDOGREL | 56.20 |
| BISOPROLOL | 52.70 |
| ESOMEPRAZOLE | 50.80 |
| OLMESARTAN | 50.50 |
| CHOLECALCIFEROL | 39.50 |
| DUTASTERIDE | 65.40 |

DDD: defined daily dose.

ATC: Anatomical Therapeutic Chemical classification. Data are from The Medicines Utilisation Monitoring Centre (2021). National Report on Medicines Use in Older Adults in Italy, 2021. Year 2019. Rome: Italian Medicines Agency AIFA. Last access: March 07, 2023 from https://www.aifa.gov.it/documents/20142/1577699/OsMed_Farmaci_anziani_13.10.2021.pdf.
